# Effect of Groundwater Iron on Residual Chlorine in Water Treated with Sodium Dichloroisocyanurate Tablets in Rural Bangladesh

**DOI:** 10.4269/ajtmh.16-0954

**Published:** 2018-02-12

**Authors:** Abu Mohd. Naser, Eilidh M. Higgins, Shaila Arman, Ayse Ercumen, Sania Ashraf, Kishor K. Das, Mahbubur Rahman, Stephen P. Luby, Leanne Unicomb

**Affiliations:** 1 International Centre for Diarrhoeal Disease Research, Bangladesh (icddr,b), Dhaka, Bangladesh;; 2 Department of Environmental Health Sciences, Rollins School of Public Health, Emory University, Atlanta, Georgia;; 3 Division of Epidemiology, School of Public Health, University of California, Berkeley, California;; 4 Stanford Woods Institute for the Environment and Freeman Spogli Institute for International Studies, Stanford University, Stanford, California

## Abstract

We assessed the ability of sodium dichloroisocyanurate (NaDCC) to provide adequate chlorine residual when used to treat groundwater with variable iron concentration. We randomly selected 654 tube wells from nine subdistricts in central Bangladesh to measure groundwater iron concentration and corresponding residual-free chlorine after treating 10 L of groundwater with a 33-mg-NaDCC tablet. We assessed geographical variations of iron concentration using the Kruskal–Wallis test and examined the relationships between the iron concentrations and chlorine residual by quantile regression. We also assessed whether user-reported iron taste in water and staining of storage vessels can capture the presence of iron greater than 3 mg/L (the World Health Organization threshold). The median iron concentration among measured wells was 0.91 (interquartile range [IQR]: 0.36–2.01) mg/L and free residual chlorine was 1.3 (IQR: 0.6–1.7) mg/L. The groundwater iron content varied even within small geographical regions. The median free residual chlorine decreased by 0.29 mg/L (95% confidence interval: 0.27, 0.33, *P* < 0.001) for every 1 mg/L increase in iron concentration. Owner-reported iron staining of the storage vessel had a sensitivity of 92%, specificity of 75%, positive predictive value of 41%, and negative predictive value of 98% for detecting > 3 mg/L iron in water. Similar findings were observed for user-reported iron taste in water. Our findings reconfirm that chlorination of groundwater that contains iron may result in low-level or no residual. User reports of no iron taste or no staining of storage containers can be used to identify low-iron tube wells suitable for chlorination. Furthermore, research is needed to develop a color-graded visual scale for iron staining that corresponds to different iron concentrations in water.

## INTRODUCTION

Household chlorination is one of the most cost-effective point-of-use (POU) water treatment interventions in resource-limited settings.^1^ Chlorine treatment inactivates the vast majority of human enteric pathogens.^2,3^ Chlorine-based disinfectants leave free chlorine residual in treated water, which provides protection against further introduced micro-organisms.^4^ The Centers for Disease Control and Prevention (CDC) recommends residual-free chlorine levels between 0.2 and 2 mg/L to ensure adequate disinfection and residual protection.^5^

Groundwater is used for drinking purposes in many countries and often contains reduced forms of iron, arsenic, manganese, and sulfur. These reduced chemicals react with chlorine-based disinfectants and increase the chlorine demand.^6^ The effectiveness of chlorine-based disinfectants in providing the targeted levels of residual-free chlorine in treated groundwater therefore varies with the chemical makeup of the groundwater aquifer.^7^ Efforts to use chlorine-based disinfectants such as calcium hypochlorite and sodium hypochlorite to treat groundwater were unsuccessful in Bangladesh,^8,9^ likely because of the interference with groundwater chemicals.

Although several groundwater chemicals react with chlorine-based disinfectants, dissolved iron in groundwater often correlates with high levels of other dissolved cations^10^ and could, therefore, be a useful proxy for overall chlorine demand from inorganic cations. The ferrous form of iron is commonly present in groundwater in Bangladesh^7^ and elsewhere^11^ because of anoxic conditions,^12^ which contributes to the redox potential of groundwater.^13^ In the presence of chlorine, the ferrous form is oxidized to the ferric form, and the chlorine residual is consumed in the process.^14^ This phenomenon has been observed in several studies of water distribution systems. Free residual chlorine decreases, while water flows through distribution systems comprised iron pipes,^15,16^ and the loss of residual is more pronounced for unlined iron pipes.^17^ Similarly iron present in groundwater decreases the concentration of chlorine residual.^18^ Such reduction in chlorine residual weakens the protection against microbial contamination.^18–21^ Therefore, chlorine disinfection is not recommended for groundwater with high iron content unless the iron is removed.

A country-wide groundwater survey in Bangladesh (British Geological Survey [BGS]-Department of Public Health Engineering) demonstrated the regional and geographical variation in iron concentration.^22^ Although the survey suggested that several regions had a relatively low groundwater iron content for which chlorination may be a feasible water disinfection method, there may be a local (e.g., village level) variation whereby wells with high iron concentration are found within relatively low iron areas. Large-scale water chlorination projects may fail if such variation of groundwater iron concentration is not taken into account.

Directly measuring iron concentration in groundwater may not be logistically or financially feasible at scale, so exploring the use of proxy indicators such as user self-reports of iron taste and staining on water storage containers may guide groundwater chlorination recommendations. Previous research suggests that high groundwater iron concentrations can be detected by taste and staining on containers.^23^ Water with an iron taste may be noticeable by consumers if the iron concentration is > 0.3 mg/L.^24^ Reddish-brown rust-colored iron stains on storage containers can occur when iron is exposed to the oxygen in air. Concentrations as low as 0.3 mg/L may cause a reddish-brown stain on utensils. The World Health Organization (WHO) recommends an acceptable iron concentration between 0.3 and 3 mg/L based on the taste and appearance of water.^25^

We conducted a study in three districts of rural Bangladesh with relatively low groundwater iron where a large randomized-controlled trial (water quality, sanitation, and hand washing [WASH] Benefits, http://www.washbenefits.net/) of water, sanitation, hygiene, and nutrition interventions including drinking water treatment with sodium dichloroisocyanurate (NaDCC) tablets was implemented.^26^ This study was conducted before trial implementation to screen sites for the WASH Benefits Bangladesh trial. Our objectives were to assess whether 1) small-scale geographic variation of iron concentration may exist in these relatively low groundwater iron areas, 2) NaDCC tablets provide the recommended chlorine residual when used to chlorinate groundwater with varying iron concentration, and 3) user-reported iron taste and iron staining on storage containers are accurate proxy indicators of high groundwater iron concentration.

## METHODS

### Study sites and sampling strategy.

The study was conducted between June and November 2012, in the period before enrolling households into the WASH Benefits trial.^26^ The sampling strategy presented here was developed to determine groundwater iron concentrations and chlorine efficacy in the region to guide site selection for the WASH Benefits trial enrollment. We conducted the study in the Gazipur, Mymensingh, and Tangail districts of central Bangladesh, where average iron concentrations were previously reported as relatively low compared with other regions of Bangladesh by the BGS.^26,27^ The BGS data suggested that the median groundwater iron concentration in Bangladesh (*N* = 3,530 tube wells) was 1.1 mg/L (interquartile range [IQR]: 0.15–4.57) and the median iron concentration in the three districts where the WASH Benefits study was conducted (*N* = 243 tube wells) was 0.43 mg/L (IQR: 0.02–3.07). The relatively low-iron region was purposefully chosen for the WASH Benefits trial to maximize the effectiveness of the chlorine-based water treatment.^28^

**Figure 1.**
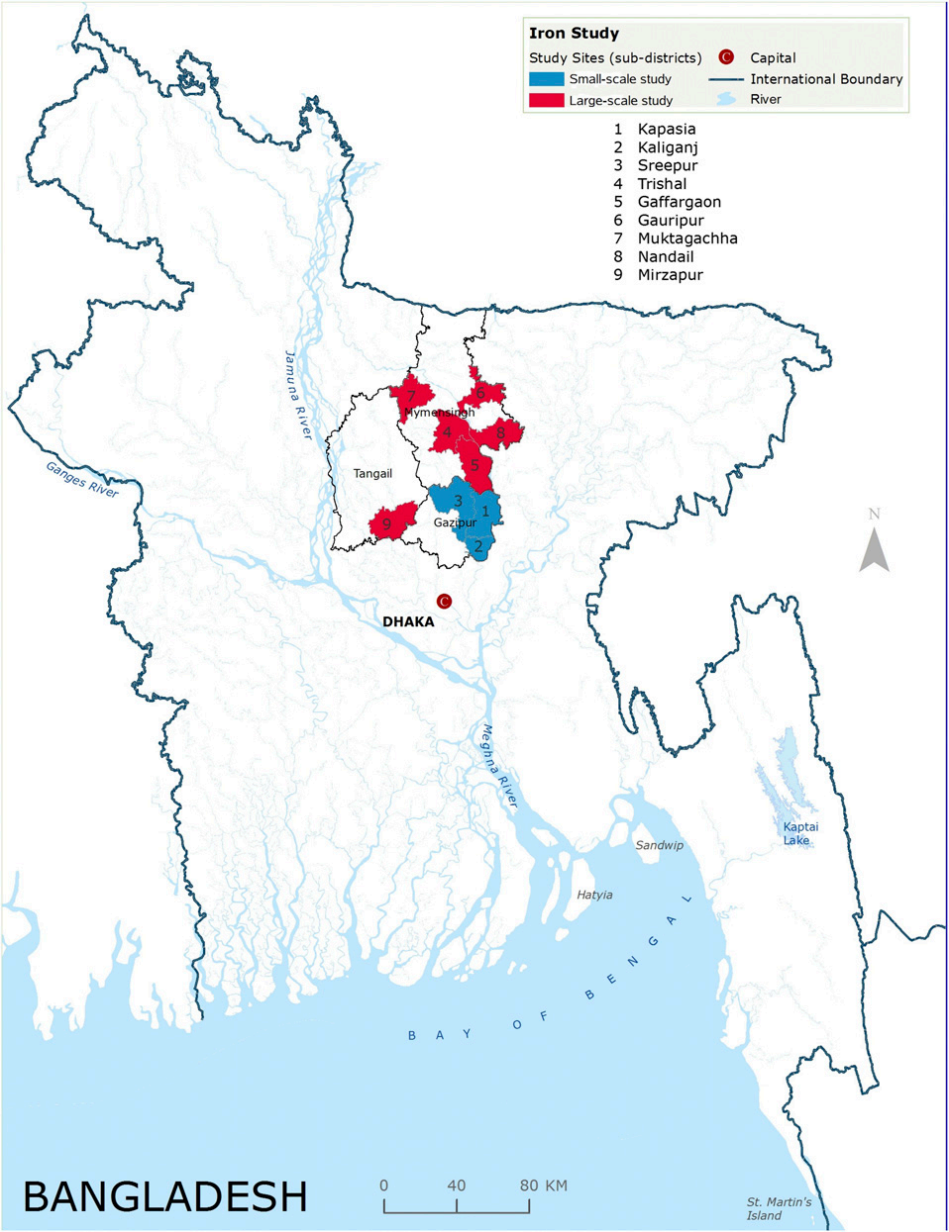
Study sites for the small- and large-scale iron study.

**Figure 2.**
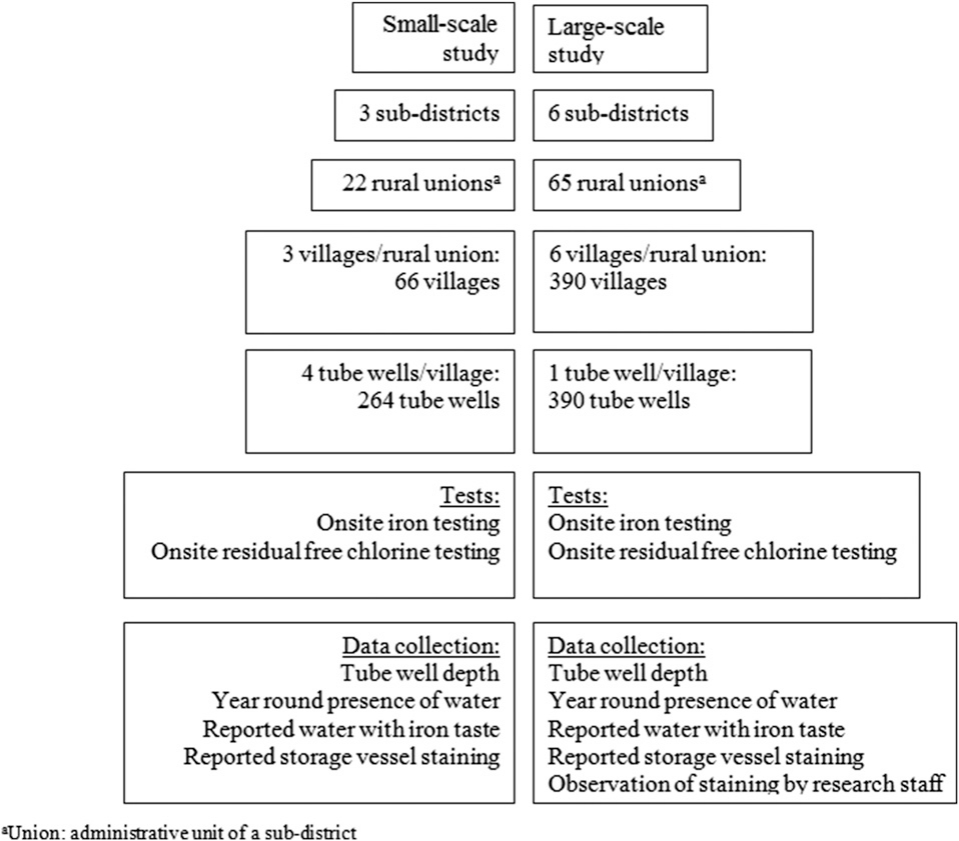
Study organizational diagram and sampling strategies.

The groundwater iron exploration study was conducted at two scales. Village level iron variability was examined by a small-scale study and subdistrict level iron variation was assessed by a large-scale study (Figure 1). In the small-scale study, research staff randomly selected three villages from each of the 22 rural unions (administrative unit of a subdistrict, comprised several villages) in three subdistricts of the Gazipur district (Figures 1 and 2). They divided each selected village into four regions after discussion with communities and identified one tube well from the approximate central point of each region (*N* = 264 tube wells). In the large-scale study, research staff randomly selected six villages from each of the 65 unions in six subdistricts of the Mymensingh and Tangail districts. In each selected village, they identified one tube well from the approximate central point (*N* = 390 tube wells).

### Data collection and water testing.

Research staff identified owners of the selected tube wells to test their tubewell water and collect well-related information. They recorded information on the tubewell depth, year-round water availability, and owners’ perception of iron taste in tubewell water and storage vessel staining. They asked “yes–no” questions to all owners on whether they felt there was an iron taste in their well drinking water and whether they observed iron staining on their water storage vessels. In the large-scale study, trained research staff also visually observed reddish-brown staining inside storage containers to confirm owners’ reports of staining. Research staff underwent field-based training where they visually inspected a number of stained-storage containers before the large-scale study.

Iron concentration in water was measured onsite using a Hach Pocket Colorimeter II for Iron (FerroVer^®^; Hach, Loveland, CO; detection range: 0.02–5.00 mg/L). Research staff then collected a separate 10-L tubewell water sample and added one 33-mg-NaDCC tablet, calculated to provide an initial free chlorine dose of 2 mg/L. After 30 minutes, residual-free chlorine was measured using a Hach Pocket Colorimeter II for chlorine (Hach; detection range: 0.02–2.00 mg/L, precision: ±0.05 mg/L). If iron concentration was detected in the upper limit (≥ 5 mg/L) and the residual chlorine concentration was less than 0.2 mg/L after adding a 33-mg-NaDCC tablet, they added an additional 33-mg-NaDCC tablet. The 30-minute period was selected to represent the CDC-recommended time window for effective disinfection after adding NaDCC; this time period is sufficient for the rapid oxidation reactions between inorganic cations and free chlorine to occur.^5^

### Statistical analysis.

We calculated the median and IQR of tubewell water iron concentration and residual-free chlorine in NaDCC-treated groundwater. We calculated the proportions of 1) tube wells with iron concentration greater than the WHO standard of 3 mg/L and 2) treated water samples with free residual chlorine less than the CDC-recommended concentration of 0.2 mg/L. We also calculated the proportion of respondents reporting the presence of iron taste in water from the well and staining of storage containers where the well water is stored.

We examined the geographic variation in iron concentration between wells in the same village by calculating the intra-class correlation at the village level using the small-scale study data and compared differences in median iron concentration at the village and union levels using the Kruskal–Wallis one-way analysis of variance. We plotted the union-level median iron concentration to visualize the variation across unions. We determined the factors associated with median iron concentration in tubewell water and free residual chlorine in treated water using quantile regression models. To assess the accuracy of the proxy measures of iron presence, we calculated the sensitivity, specificity, positive predictive value, and negative predictive value of self-reported iron taste in tubewell water and staining of storage vessels against measured iron concentrations ≥ 3 mg/L.

### Ethical consideration.

The assessment for groundwater iron was part of the screening eligibility for the WASH Benefits study and was approved by the ethical review committee of icddr,b as part of the WASH Benefits study. Verbal consent from all respondents was taken before participation in the study.

## RESULTS

**Figure 3.**
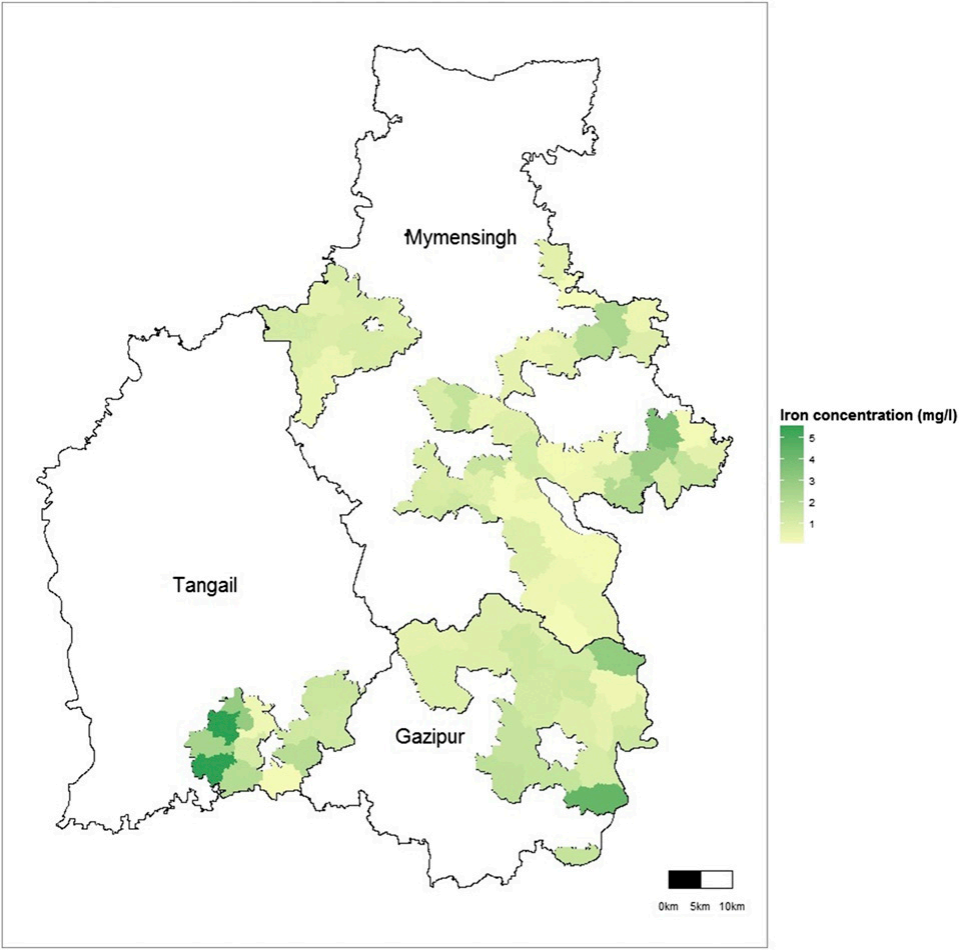
Variation of the median well water iron concentration across unions.

The median iron concentration of the 654 tested wells was 0.91 mg/L (IQR: 0.36–2.01 mg/L; Table 1), and 18% (117/654) of wells had iron concentrations greater than 3 mg/L (range: 7–38% at the subdistrict level; Table 1). Among 22 unions in the small-scale study, 15 unions had at least one well with iron concentrations greater than 3 mg/L. Median iron concentration of tube wells varied within small geographical areas with significant variation between villages in the same union (*P* = 0.008) and between unions (*P* = 0.0001). In the Kaliganj subdistrict, the union-level median iron concentration varied from 1.3 to 4.3 mg/L; in Kapasia from 0.4 to 3.0 mg/L, and in Sreepur from 0.6 to 1.8 mg/L (Figure 3, Table 2). When we considered a village as a cluster, the intra-cluster correlation coefficient within a union ranged from 0 to 0.54 (Table 2). In the larger-scale study, the subdistrict level median iron concentration varied from 0.31 to 1.94 mg/L (*P* = 0.0001; Table 1). The median tubewell depth was 150 ft (range: 12–550 ft). Iron concentration was inversely associated with the reported depth of tube wells—there was a 0.20 mg/L (95% confidence interval [CI]: −0.30, −0.10, *P* < 0.001) decrease in median iron concentration for each 100 feet increase in well depth. Iron concentration was not associated with year-round presence of water in tube wells (Table 3).

**Table 1:** Iron concentration in tubewell water and residual-free chlorine levels in treated water 30 minutes after adding a 33-mg-sodium dichloroisocyanurate tablet to 10 L of water in the small- and large-scale study

Study	Subdistrict	Iron concentration		Residual-free chlorine	
		Median (IQR)	Greater than 3 mg/L, *n* (%)	Median (IQR)	Less than 0.2 mg/L, *n* (%)
Small-scale study	Kaligonj (*N* = 48)	1.4 (0.7–2.1)	10 (21)	1.1 (0.3–1.6)	11 (23)
	Kapasia (*N* = 120)	1.2 (0.52–2.9)	28 (23)	1.4 (0.5–1.6)	21 (18)
	Sreepur (*N* = 96)	1.12 (0.46–2.1)	18 (19)	1.4 (0.77–1.7)	11 (11)
Large-scale study	Gafaorgaon (*N* = 84)	0.31 (0.17–0.62)	3 (4)	1.4 (0.92–1.7)	8 (80)
	Nandail (*N* = 66)	0.61 (0.27–2.25)	15 (23)	1 (0.25–1.6)	14 (21)
	Muktagacha (*N* = 60)	0.81 (0.42–1.47)	4 (7)	1.4 (1–1.7)	5 (8)
	Trishal (*N* = 60)	1 (0.46–1.77)	7 (12)	1.3 (0.9–1.6)	8 (13)
	Gouripur (*N* = 54)	0.73 (0.25–1.62)	7 (13)	1.8 (0.87–2.1)	4 (7)
	Mirzapur (*N* = 66)	1.94 (0.6–5)	25 (38)	0.9 (0.07–1.6)	21 (32)
Both studies	Total (*N* = 654)	0.91 (0.36–2)	117 (18)	1.3 (0.6–1.7)	103 (16)

IQR = interquartile range.

**Table 2:** Groundwater iron variation at small geographical region (union-level) and residual-free chlorine levels in treated water in the small-scale study

Subdistrict	Union*	Iron concentration			Residual-free chlorine	
		Median (IQR)†	Greater than 3 mg/L, *n* (%)	Intra-cluster correlation coefficient	Median (IQR)	Less than 0.2 mg/L, *n* (%)
Kaligonj (*N* = 48)‡	Kaligonj	1.6 (0.4–2.0)	2 (17)	0.04	1.3 (0.52–1.64)	2
	Muktarpur	4.3 (1.1–5.5)	8 (67)	0	0.14 (0.06–1.05)	8
	Nagari	1.3 (0.6–1.9)	0	0.3	1.26 (0.81–1.64)	0
	Tumulia	1.3 (0.8–1.6)	0	0.08	1.13 (0.50–1.75)	1
Kapasia (*N* = 120)‡	Barishaba	0.4 (0.3–1.1)	0	0	1.63 (1.54–1.79)	0
	Chandpur	1.6 (0.9–2.4)	2 (17)	0.24	0.26 (0.14–1.07)	4
	Durgapur	1.1 (0.5–5)	4 (33)	0.25	1.41 (0.49–1.57)	3
	Ghagotia	1.1 (0.5–1.5)	0	0.28	1.46 (0.21–1.61)	3
	Karihata	0.6 (0.3–3.7)	4 (33)	0.06	1.44 (1.08–1.61)	2
	Rayed	1.3 (0.6–2.9)	2 (17)	0.54	1.15 (0.54–1.65)	3
	Sanmania	1.4 (0.7–2.7)	3 (25)	0.09	1.64 (0.86–1.80)	0
	Singasree	1.2 (0.8–3.6)	5 (42)	0.45	1.60 (1.33–1.71)	1
	Toke	3.0 (1.3–3.6)	6 (50)	0.06	0.61 (0.05–1.34)	4
	Taragaon	0.9 (0.5–1.7)	2 (17)	0.12	1.38 (0.89–1.47)	0
Sreepur (*N* = 96)‡	Barami	1.4 (0.4–2.3)	0	0.12	1.56 (0.25–1.65)	3
	Gazipur	0.6 (0.3–1.1)	0	0.13	1.58 (1.32–1.79)	0
	Gosinga	1.2 (0.5–1.4)	0	0	1.11 (0.67–1.52)	0
	Kaoraid	0.9 (0.4–1.9)	2 (17)	0.36	1.11 (0.50–1.45)	2
	Mawna	0.8 (0.2–4.2)	4 (33)	0.42	1.67 (1.36–1.91)	0
	Prohladpur	1.8 (0.6–4.7)	4 (33)	0	1.31 (0.49–1.69)	2
	Rajbari	1.6 (1.0–3.6)	3 (33)	0	1.38 (0.59–1.74)	1
	Telihati	0.9 (0.5–5.0)	5 (42)	0.21	1.36(0.29–1.68)	3

* Union: administrative unit of a subdistrict; for each union, 12 tube wells were sampled.

† Interquartile range.

‡ Twelve tube wells tested in each village.

**Table 3:** Predictors of iron concentration in tubewell water and residual-free chlorine in treated water

	Co-efficient	95% confidence interval	*P* value
Factors affecting iron concentration*			
Tubewell depth (feet)	−0.002	−0.003, −0.001	< 0.001
Tubewell location (subdistrict)	0.074	0.035, 0.114	< 0.001
Year round presence of water in the tube well†	0.164	−0.130, 0.457	0.274
Factors affecting residual-free chlorine*			
Tubewell water iron concentration (mg/L)	−0.299	−0.332, −0.265	< 0.001

* Quantile regression models.

† Binary variable (yes/no).

The median residual-free chlorine 30 minutes after adding one 33-mg-NaDCC tablet to 10-L-well water samples was 1.3 mg/L (IQR: 0.6–1.7 mg/L), and 16% of samples (103/654) had residual-free chlorine < 0.2 mg/L (Table 1). Residual-free chlorine in treated water was inversely associated with iron concentration—a 0.29-mg/L (95% CI: −0.33, −0.27 *P* < 0.001) residual-free chlorine reduction was associated with every 1 mg/L increase in iron concentration (Table 3).

Tubewell water iron taste was reported by 42% (274/653) of well owners and staining of storage containers by 37% (144/390) of owners. Research staff observed staining of the storage vessels in 35% (138/390) of households. The correlation between owner-reported and observed iron staining of the vessels was 0.97 (*P* < 0.0001). Owner-reported iron taste had a sensitivity of 84%, specificity of 67%, positive predictive value of 36%, and negative predictive value of 95% for detecting iron greater than 3 mg/L. Owner-reported staining of storage containers had a sensitivity of 92%, specificity of 75%, positive predictive value of 41%, and negative predictive value of 98% for detecting iron greater than 3 mg/L.

## DISCUSSION

The region selected for this study was reported by the BGS in 2001 to have low average iron concentration; we detected a similar median iron concentration (0.9 mg/L) in this study conducted approximately 10 years later, surveying a larger number of tube wells within selected sites (*N* = 654) than the BGS (*N* = 84). Even though the sites were considered to have a low average iron concentration, we found variation across small geographical units (i.e., villages) and large geographical units. In our large-scale study, we found that between 4% and 38% of wells had an iron concentration greater than 3 mg/L. Based on these findings, we did not recommend the Mirzapur subdistrict as a potential site for the WASH Benefits Bangladesh study as 38% of wells in this subdistrict had an iron concentration greater than 3 mg/L. The variability in iron concentration was similar to that found in a study conducted in a smaller area of northwestern Bangladesh.^23^ Local variability can be explained by geochemical properties of groundwater aquifers.^28^ The source of dissolved ions in groundwater includes mineral accumulations in rocks near the land surface;^11^ thus iron-containing materials are usually present in shallow aquifers. Moreover, dissolved organic matter in shallow aquifers in Bangladesh can act as a substrate for iron-reducing bacteria, enhancing iron concentration in superficial layers. These factors may explain the inverse relationship between the iron concentration and estimated tubewell depth.^23,29^

We identified a statistically significant inverse association between free residual chlorine and iron concentration in groundwater, which confirms findings from several studies that the high water iron content reduces the effectiveness of chlorination. In our study site with generally average low groundwater iron content, almost one in five tube wells had an iron concentration greater than 3 mg/L, and optimal residual was not achieved for one in six tube wells tested. Elsewhere in Bangladesh, the average iron concentration is often higher,^27^ and thus failure to achieve optimal chlorine residual is likely.^23^ A high initial chlorine dose would be required to achieve residual > 0.2 mg/L for wells with high iron concentration, which may preclude acceptability and uptake because of unpleasant taste and odor of treated water.^30^

Given the geographical variability in groundwater iron concentrations, the need to identify tube wells with high iron content during large-scale chlorination interventions presents an obstacle to using chlorine to treat groundwater. Measuring iron concentration for each well to recommend a targeted chlorine dose is not feasible. In our study, owner-reported iron taste and staining of water storage containers had high sensitivity, specificity, and negative predictive values but low positive predictive values. Nevertheless, iron staining of the storage containers was a better proxy than iron taste in water to detect high-iron wells. The high sensitivity of these proxies suggests that household members can accurately identify tube wells with iron concentration > 3 mg/L 84% of the time; similar findings have been reported by others.^23^ The high negative predictive values suggest that among wells that are reported by users to be iron free, a high proportion (95%) is likely to have low iron concentrations at the time of testing. Therefore, areas where owners report no iron taste or staining of storage vessels may indicate those where chlorination is appropriate. We advise programs promoting POU chlorination to target households that use wells where owners report no iron taste or staining of storage vessels. In contrast, the low positive predictive values suggest that among wells where users report high iron, only a small proportion (36%) truly has high concentrations (> 3 mg/L) at the time of testing.

Our study had certain limitations. We measured the iron concentration of tube wells at a single time point. However, seasonal variation in iron concentration has been observed in shallow and deep groundwater aquifers in Bangladesh,^31^ which may affect our sensitivity and specificity estimates. It is likely that owner-reported iron taste in tubewell water reflects the iron content over a longer recall period rather than at the time of testing. Therefore, such proxy information may be a better metric than a one-time iron measurement to identify wells where the high-iron content in the long run will render chlorination ineffective. Our research staff visually confirmed the presence/absence of iron staining of the storage containers, which does not provide quantitative information about the iron concentration in water. Iron corrosion scales to quantify the extent of corrosion in water distribution systems^32^ and commercial iron testing strips with color charts have been useful in other settings to assess the concentration of iron in water. A similar scale can be developed to quantify the extent of staining on containers. For example, a visual staining scale with different color grades corresponding to different categories of iron concentrations can allow semiquantitative assessment of iron concentration inwater.

Although we only tested iron, other groundwater inorganic cations interact with chlorine in a similar fashion. Nevertheless, there are positive correlations between groundwater iron concentration and other chemicals such as arsenic and manganese; our iron measurements therefore may have been indicative of the presence of a range of chemicals exerting chlorine demand.^33^

Our data reconfirm the findings of other studies that chlorination is not a practical water treatment method in settings with high groundwater iron concentration. When iron testing is not feasible, we recommend using owner-reports of absence of iron taste in groundwater or absence of storage containers’ staining to identify tube wells suitable for chlorination.

## References

[1] Clasen T Cairncross S Haller L Bartram J Walker D, 2007 Cost-effectiveness of water quality interventions for preventing diarrhoeal disease in developing countries. J Water Health 5: 599–608. 17878570 10.2166/wh.2007.010

[2] Clasen T Edmondson P, 2006 Sodium dichloroisocyanurate (NaDCC) tablets as an alternative to sodium hypochlorite for the routine treatment of drinking water at the household level. Int J Hyg Environ Health 209: 173–181. 16387550 10.1016/j.ijheh.2005.11.004

[3] Arnold BF Colford JM Jr, 2007 Treating water with chlorine at point-of-use to improve water quality and reduce child diarrhea in developing countries: a systematic review and meta-analysis. Am J Trop Med Hyg 76: 354–364. 17297049

[4] Sobsey MD Stauber CE Casanova LM Brown JM Elliott MA, 2008 Point of use household drinking water filtration: a practical, effective solution for providing sustained access to safe drinking water in the developing world. Environ Sci Technol 42: 4261–4267. 18605542 10.1021/es702746n

[5] Centers for Disease Control and Prevention, 2014 *Free Chlorine Testing*. Available at: https://www.cdc.gov/safewater/chlorine-residual-testing.html. Accessed January 24, 2018.

[6] Lippy EC, 1986 Chlorination to prevent and control waterborne diseases. J Am Water Works Assoc 78: 49–52.

[7] Hossain MD Huda MK, 1997 Study of iron content in groundwater of Bangladesh. J Civ Eng 25: 171–179.

[8] Ferguson AS Mailloux BJ Ahmed KM van Geen A McKay LD Culligan PJ, 2011 Hand-pumps as reservoirs for microbial contamination of well water. J Water Health 9: 708–717. 22048430 10.2166/wh.2011.106 PMC5920553

[9] Luby S Islam MS Johnston R, 2006 Chlorine spot treatment of flooded tube wells, an efficacy trial. J Appl Microbiol 100: 1154–1158. 16630017 10.1111/j.1365-2672.2006.02940.x

[10] Dowling CB Poreda RJ Basu AR Peters SL Aggarwal PK, 2002 Geochemical study of arsenic release mechanisms in the Bengal Basin groundwater. Water Resour Res 38: 12-1–12-18.

[11] Hem JD, 1985. Study and Interpretation of the Chemical Characteristics of Natural Water. Alexandria, VA: Department of the Interior, US Geological Survey.

[12] Stefan MI, 2017 *Advanced Oxidation Processes for Water Treatment: Fundamentals and Applications*. London, United Kingdom: IWA Publishing.

[13] Hem JD, 1960 *Restraints on Dissolved Ferrous Iron Imposed by Bicarbonate Redox Potential, and pH*. Washington, DC: US Government Printing Office.

[14] National Research Council, Safe Drinking Water Committee, 1977 *Drinking Water and Health*. Washington, DC: The National Academy of Sciences.

[15] Al-Jasser A, 2007 Chlorine decay in drinking-water transmission and distribution systems: pipe service age effect. Water Res 41: 387–396. 17140619 10.1016/j.watres.2006.08.032

[16] Hallam N West JR Forster C Powell J Spencer I, 2002 The decay of chlorine associated with the pipe wall in water distribution systems. Water Res 36: 3479–3488. 12230193 10.1016/s0043-1354(02)00056-8

[17] Sarin P Snoeyink V Bebee J Jim K Beckett M Kriven W Clement J, 2004 Iron release from corroded iron pipes in drinking water distribution systems: effect of dissolved oxygen. Water Res 38: 1259–1269. 14975659 10.1016/j.watres.2003.11.022

[18] Gordon G Cooper WJ Rice RG Pacey GE, 1988 Methods of measuring disinfectant residuals. J Am Water Works Assoc 80: 94–108.

[19] Clark RM Yang YJ Impellitteri CA Haught RC Schupp DA Panguluri S Krishnan ER, 2010 Chlorine fate and transport in distribution systems: experimental and modeling studies. J Am Water Works Assoc 102: 144–155.

[20] Islam N Sadiq R Rodriguez MJ, 2013 Optimizing booster chlorination in water distribution networks: a water quality index approach. Environ Monit Assess 185: 8035–8050. 23532783 10.1007/s10661-013-3153-z

[21] Aieta EM Berg JD, 1986 A review of chlorine dioxide in drinking water treatment. J Am Water Works Assoc 78: 62–72.

[22] Kinniburgh D Smedley P, 2001 Arsenic contamination of groundwater in Bangladesh. Kinniburgh DG, Smedley PL, eds. *British Geological Survey Report WC/00/19*. Keyworth, United Kingdom: British Geological Survey.

[23] Merrill R Labrique A Shamim A Schulze K Christian P West K, 2010 Elevated and variable groundwater iron in rural northwestern Bangladesh. J Water Health 8: 818–825. 20705991 10.2166/wh.2010.144

[24] WHO, 2003 *Iron in Drinking-Water: Background Document for Development of WHO Guidelines for Drinking-Water Quality* Available at: http://www.who.int/water_sanitation_health/dwq/chemicals/iron.pdf. Accessed May 18, 2017.

[25] WHO, 2011 *Guidelines for Drinking-Water Quality*, 4th edition. Available at: http://whqlibdoc.who.int/publications/2011/9789241548151_eng.pdf. Accessed July 8, 2016.

[26] Arnold BF Null C Luby SP Unicomb L Stewart CP Dewey KG Ahmed T Ashraf S Christensen G Clasen T, 2013 Cluster-randomised controlled trials of individual and combined water, sanitation, hygiene and nutritional interventions in rural Bangladesh and Kenya: the WASH benefits study design and rationale. BMJ Open 3: e003476. 10.1136/bmjopen-2013-003476 PMC3758977 23996605

[27] British Geological Survey, 2001 *Groundwater Quality: Bangladesh 2001* Available at: https://www.bgs.ac.uk/downloads/start.cfm?id=1277. Accessed August 5, 2017.

[28] Nelson D, 2002 *Natural Variations in the Composition of Groundwater*. Presented at Groundwater Foundation Annual Meeting, November 2012, Eugene, Oregon.

[29] Mladenov N Zheng Y Miller MP Nemergut DR Legg T Simone B Hageman C Rahman MM Ahmed KM McKnight DM, 2009 Dissolved organic matter sources and consequences for iron and arsenic mobilization in Bangladesh aquifers. Environ Sci Technol 44: 123–128. 10.1021/es901472g 20039742

[30] Flanagan S Meng X Zheng Y, 2013 Increasing acceptance of chlorination for household water treatment: observations from Bangladesh. Waterlines 32: 125–134.

[31] Dhar R Zheng Y Stute M Van Geen A Cheng Z Shanewaz M Shamsudduha M Hoque M Rahman M Ahmed K, 2008 Temporal variability of groundwater chemistry in shallow and deep aquifers of Araihazar, Bangladesh. J Contam Hydrol 99: 97–111. 18467001 10.1016/j.jconhyd.2008.03.007 PMC2605690

[32] Yang F Shi B Gu J Wang D Yang M, 2012 Morphological and physicochemical characteristics of iron corrosion scales formed under different water source histories in a drinking water distribution system. Water Res 46: 5423–5433. 22882957 10.1016/j.watres.2012.07.031

[33] Ayotte JD Nielsen MG Robinson GR Jr Moore RB, 1999 *Relation of Arsenic, Iron, and Manganese in Ground Water to Aquifer Type, Bedrock Lithogeochemistry, and Land Use in the New England Coastal Basins*. Pembroke, NH: US Department of the Interior, US Geological Survey.

